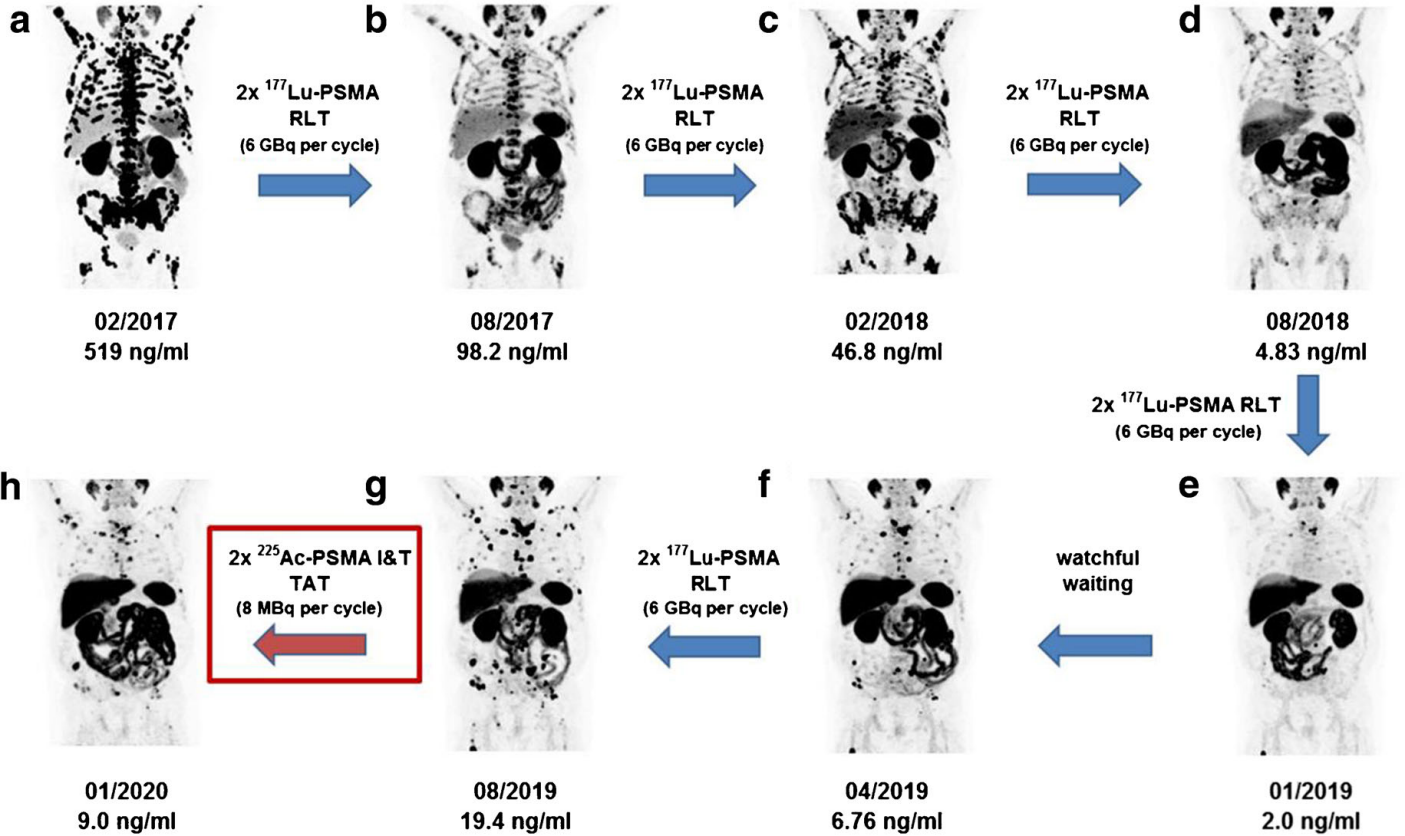# Response to ^225^Ac-PSMA-I&T after failure of long-term ^177^Lu-PSMA RLT in mCRPC

**DOI:** 10.1007/s00259-020-05023-2

**Published:** 2020-09-22

**Authors:** Harun Ilhan, Astrid Gosewisch, Guido Böning, Friederike Völter, Mathias Zacherl, Marcus Unterrainer, Peter Bartenstein, Andrei Todica, Franz Josef Gildehaus

**Affiliations:** 1 Department of Nuclear Medicine, University Hospital, LMU Munich, Munich, Germany; 2 Department of Radiology, University Hospital, LMU Munich, Munich, Germany

Radioligand therapy (RLT) using ^177^Lu-PSMA ligands is highly effective in metastatic castration-resistant prostate cancer (mCRPC); however, failure of ^177^Lu-PSMA RLT remains challenging as RLT already represents last-line treatment.

The α-emitter ^225^Ac provides higher biological effectiveness compared with ^177^Lu [1]. Several centers reported remarkable response after PSMA-targeted alpha therapy (TAT) using ^225^Ac-PSMA-617 after failure of ^177^Lu-PSMA RLT [2, 3]. Here we present encouraging response to TAT in a patient with advanced mCRPC showing progression after long-term ^177^Lu-PSMA RLT (10 cycles). PSA values are provided under the date of each PSMA-PET MIP image (A–B using ^68^Ga-PSMA-11 and E–H using ^18^F-PSMA-1007). The patient was referred for RLT after radical prostatectomy and radiotherapy in 2005, and anti-hormonal therapy started in 2013 due to biochemical progression. Further progression was observed in February 2017 (A) after 2nd-line anti-hormonal therapy from 2015 to 2016, ^223^Ra-Dichloride in 2016, and docetaxel chemotherapy from 2016 to 2017. Two cycles of ^177^Lu-PSMA-617 were highly effective (B). PSA was still decreasing after two additional ^177^Lu-PSMA-617 cycles despite increasing PSMA-ligand uptake in PSMA-PET (C). Maintenance therapy using ^177^Lu-PSMA-617 was continued until January 2019 with further response (D and E); however, disease progression occurred after watchful waiting and two cycles of ^177^Lu-PSMA-I&T (F and G). The patient then received two cycles of ^225^Ac-PSMA-I&T and showed encouraging response (H). The main TAT-related side effect was grade 2 xerostomia (grade 2), which was already preexisting after 10 cycles of RLT. No TAT-related grade 3/4 hematological side effects were noted. Further cycles are planned but were suspended due to the COVID-19 crisis upon patient’s request.

Different approaches including tandem therapy with ^177^Lu or de-escalating doses during consolidation have been proposed for TAT as a trade-off between therapeutic efficacy and tolerable side effects [2, 4], and further studies investigating ^225^Ac-PSMA remain highly important for prostate cancer theranostics.